# The lived experience of people with upper limb absence living in Uganda: A qualitative study

**DOI:** 10.4102/ajod.v11i0.890

**Published:** 2022-05-20

**Authors:** Dafne Zuleima Morgado Ramirez, Brenda Nakandi, Robert Ssekitoleko, Louise Ackers, Erisa Mwaka, Laurence Kenney, Cathy Holloway, Maggie Donovan-Hall

**Affiliations:** 1 Interaction Centre, Department of Computer Science, Faculty of Engineering, University College London, London, United Kingdom; 2 Global Disability Innovation Hub, London, United Kingdom; 3 Biomedical Engineering Unit, Department of Physiology, School of Biomedical Sciences, College of Health Sciences, Makerere University, Kampala, Uganda; 4 School of Health and Society, University of Salford, Salford, United Kingdom; 5 Department of Anatomy, College of Health Sciences, Makerere University, Kampala, Uganda; 6 Centre for Health Sciences Research, University of Salford, Salford, United Kingdom; 7 School of Health Sciences, Faculty of Life and Environmental Sciences, University of Southampton, Southampton, United Kingdom

**Keywords:** ableism, amputation, disability, lived experience, psychosocial, upper limb

## Abstract

**Background:**

The impact of upper limb absence on people’s lived experiences is understudied, particularly in African countries, with implications for policy and service design.

**Objectives:**

The objective of this study was to explore the lived experiences of people with upper limb absence (PWULA) living in Uganda.

**Method:**

Informed by preliminary work, we designed a qualitative study employing semi-structured interviews to understand the experience of living with upper limb absence in Uganda. Seventeen adults with upper limb absence were individually interviewed and their interviews were analysed utilising thematic analysis.

**Results:**

Seven themes illustrating the impact on the individual’s life after amputation were identified and categorised into (1) living and adapting to life, (2) productivity and participation and (3) living within the wider environment. This study presents three main findings: (1) PWULA need psychological and occupational support services which are not available in Uganda, (2) PWULA want to work, but face multiple barriers to employment and has limited support, combined with the complex parenting and caring responsibilities, (3) the local Ugandan culture and social structures affect the everyday life of PWULA, both in positive and negative ways.

**Conclusion:**

This study provides information on the lived experiences of PWULA in Uganda which are lacking in the literature. People with upper limb absence face ableism and hardship underpinned by a lack of formal support structures and policies, which may in turn exacerbate the impact of upper limb absence on multiple facets of life.

## Introduction

It is estimated that globally 65 million people live with limb amputations, and 1.5 million people undergo amputations every year, with 40% being upper limb amputations (Lao et al. 2020). Two thirds of people with amputation live in low resourced settings (Lao et al. 2020) and it is estimated that 5 million of the amputee population live in Africa, of which around 25% are upper limb amputees (Lao et al. 2020). An upper limb amputation is a surgical procedure that removes a part of the upper limb (hand, forearm, upper arm, shoulder) following disease or trauma (Maduri & Akhondi 2020). In low resource settings, traumatic experiences such as road accidents and conflicts (war, civil conflicts) are common causes of upper limb amputation, as are poor access to acute medical care (Commission on the Social Determinants of Health 2008; Kenney et al. 2019).

In Uganda, functional difficulties (which can lead to disability) have been measured, but have only focused on walking difficulties and sensory impairments (Uganda Bureau of Statistics 2018, 2019). About 12.5% of the Ugandan population has at least one form of walking or sensory disability (Uganda Bureau of Statistics 2019). Unfortunately, the percentage of people with physical disabilities related to the upper limbs in Uganda is unknown, although by 1996 the number of people with limb loss was estimated to be over 5 000 (Staats 1996). However, upper limb loss is known to be common, due in part to the 20 years of civil conflict in the north, north east and western regions in Uganda that resulted in maiming of people with many ending up with limb amputations (Dolan 2009). The number of people with amputation or upper limb absence (PWULA) by 1996 was estimated to be over 5 000 (Staats 1996). Previous work has shown that there is no data published on the number of PWULA and no amputee registry in Uganda (Kenney et al. 2019). However, in the last decade, trauma accounted for the highest number of incidents of upper limb amputation in Uganda’s referral hospitals (Muhumuza & Bangirana 2015), with only two studies exploring aetiology, outcomes, experiences, challenges and prevalence of people with upper and lower limb amputation living in the Acholi sub-region of northern Uganda (Atim et al. 2020; Okello et al. 2019). Amputees in northern Uganda face inaccessible and inappropriate rehabilitation services, stigma and marginalisation (Okello et al. 2019). The prevalence of major upper and/or lower limb loss was estimated to be 11 400 people out of 1.9 million in the Acholi sub-region (Atim et al. 2020). In addition, a consultation with various stakeholders in Kampala (Kenney et al. 2019) revealed that most of limb loss in Uganda is currently due to trauma caused by road accidents, violence, fire, occupational accidents, congenital limb loss, illness and improper intravenous therapy practices. While in the Acholi region, the most common reasons for amputation are malignancy, gangrene and diabetes complications (Atim et al. 2020).

Limb loss or absence is a disability and thus the Ugandan legislation on disability is relevant to people with upper limb absence (PWULA). In 2010, a study comparing international and Ugandan disability legislation noted that there was a need for more resources for disabled people, leadership and collaboration between government, funding bodies and disabled people’s organisations (DPOs), disability awareness and training, representation from all types of disabilities, and further legislation to cater for disabled people in Uganda (Millward et al. 2005). Following this, Uganda ratified the United Nations Convention on the Rights of Persons with Disabilities followed by a report in 2016 to the UN Committee that provided a list of concerns and recommendations (Committee on the Rights of Persons with Disabilities 2016). Concerns included the prevalence of the use of derogatory language towards people with disabilities (PWDs), absence of mechanisms to consult DPOs beyond the National Council for Disability, insufficient legal protections for PWDs against discrimination and lack of mechanisms to create public awareness of stigmatising cultural practises. In 2019, the African Union analysed policy and strategy documents from Uganda, Kenya, Sierra Leone and Zambia (Lang et al. 2019) and showed that recognition of the rights of PWDs is not integrated within implementation plans, budgetary allocations, enforcement mechanisms and disaggregated management information systems. This political context sets the scene for our study because PWULA in Uganda may be affected by that lack of legal protection within an environment that is psychosocially detrimental.

About 75% of Uganda’s population lives in rural areas where 80% of households are involved in agriculture and 69% depend on subsistence farming (Uganda Bureau of Statistics 2019). Activities of daily living in these settings are done manually, for example, personal care, childcare, collecting water and firewood, digging, laundry and dishwashing, among others. Furthermore, most agriculture in Uganda is non-mechanised and thus a majority use hand-held tools to tend gardens and farms. Studies in other settings have shown that, with partial or full loss of one hand or both hands, PWULA in Uganda have difficulty performing daily life activities and thus their quality of life diminishes and their reintegration in society becomes challenging (Shahsavari et al., 2020). Additional challenges are pain (Davidson, Khor & Jones 2010; Desmond & Maclachlan 2010), infections (Ajibade, Akinniyi, & Okoye 2013), muscle contractures (Baker & Clouse 2016), social and economic discrimination (Beisland & Mersland 2014; Murphy 2005; Sood et al. 2020) and psychological issues such as post-traumatic stress disorder and depression (with women being more affected) (Armstrong et al. 2019). Barriers to economic participation are also intersectional^1^ with multidimensional poverty^2^ (Eide, Khupe & Mannan 2014; United Nations Development Programme and Oxford Poverty and Human Development Initiative 2019), gender-based violence (Guloba et al. 2018) and poor access to prosthetic devices (Lao et al. 2020) further affecting people with disability. In the Acholi region, people with major limb loss that participated in a study (Atim et al. 2020) had no access to assistive technology (45.5%), had no access to rehabilitation services (46.6%) and 9.4% reported never having accessed any type of healthcare, only 1% of surveyed individuals with limb absence had been referred to rehabilitation (Atim et al. 2020).

Research aimed at understanding the lived experience of people living with limb loss is fundamental in shaping and improving current prosthetics and orthotics services and directing future research (Dillon et al. 2019). Previous qualitative research aimed at understanding the lived experience of people with limb absence has focused mostly on lower limb and has been conducted in predominantly high resourced settings (Europe and North America) (Atim et al. 2020; Johansen et al. 2018; Ligthelm & Wright 2014; Stutts et al. 2015; Vargas et al. 2014; Woods et al. 2018). A study in the Acholi region of Uganda found that people with upper and/or lower limb absence experience stigma and marginalisation, which affect relationships and job prospects (Atim et al. 2020). We came across only one other study that explored the living experiences of individuals living with upper limb loss from sub-Saharan Africa; and that was in the Tswane region of South Africa (Ligthelm & Wright 2014). Studies from outside Africa show that PWULA have difficulty in engaging in work related activities (Johansen et al. 2018), interacting with others for intimate relationships (Stutts et al. 2015; Woods et al. 2018) and they experience a lack of coordination among teams responsible for their rehabilitation (Vargas et al. 2014).

Given the scarcity of data regarding the lived experience of PWULA in Uganda and the social value that such knowledge would have in shaping upper limb prosthetics services, this study aimed to explore the lived experiences of PWULA living in Uganda. This paper explores the experience of people living with limb loss in Uganda, regardless of their time since their amputation, looking at their environment including psychosocial aspects, participation, and culture. It therefore does not just focus on acquiring a disability and adjusting to an amputation.

## Methods

This study is part of a research programme (Kenney et al. 2018) aimed at creating fit-for-purpose upper-limb prostheses for use in low resource settings, underpinned by the user’s needs (Hayes, Buckland & Tarpey 2012). This current study reported here involved two phases and was carried out in Uganda with research partners based in Kampala. Phase 1 has been published elsewhere and consisted of scoping work adopting a ‘Patient and Public Involvement and Engagement’ (PPIE) framework which is an approach used to ensure the engagement of stakeholders that the research is intended to benefit in the design and development of the research, so as to gain a clear understanding of the environment (Kenney et al. 2019). This involved carrying out thorough scoping and exploratory work, and informal discussion visits at a range of settings in the Kampala and regional areas, including public hospitals, non-governmental rehabilitation services, orthopaedic workshops, private clinics, and companies within the field of Prosthetics and Orthotics service delivery. Phase 1 informed the development and design of the semi-structured qualitative study presented in this paper (Phase 2). Phase 2 consisted of an exploratory qualitative study employing a semi-structured interview approach to understand the experience of people living with upper limb absence in Uganda. As there is limited previous research exploring the aims of people with upper limb loss in low resource settings taking a user-led approach, an approach independent of an epistemological or theoretical framework was taken to provide maximum flexibility (Braun & Clarke 2013).

The completion of Phase 1 provided the opportunity for Ugandan and UK teams to work in collaboration and identify the needs for Phase 2 in terms of co-design approaches and for the data to be more acceptable for participants and for the local team to have a much deeper understanding of Uganda’s linguistic and cultural diversity and social issues. This involved identifying important issues regarding the need for researcher capacity building, working in collaboration to reduce appropriate written information for participants, a pragmatic recruitment approach and the development of a flexible interview schedule that could be tailored to each participant’s needs. To build capacity within the local team, a bespoke qualitative research methods’ training package was developed in collaboration with team members. This was delivered in person by a qualitative researcher, in a two day workshop for the four Ugandan team members involved in the data collection. The first day theme was to understand the aims of this study and processes of qualitative research, which involved all aspects of the research process including gaining ethical approval, carrying out ethical and responsible research and the handling of data. During the second day, the semi-structured interview schedule was studied and practised for developing interview skills. The semi-structured interview questions were revised to be socially and culturally relevant. The full interview schedule is made available in Appendix 1.

### Study population and sampling strategy

Convenience sampling was used to select PWULA who were registered with an Orthopaedic Clinic in Kampala, which was already familiar with the participants and was outside of the clinic setting (neutral and private environment for open discussions). Due to the exploratory nature of the research, a convenience sample of anyone with unilateral or bilateral limb absence, due to any cause, and any level beyond only digits (below elbow or above elbow), shoulder disarticulation and forequarter amputation, were included in this study. From Phase 1, we learned that the most culturally appropriate way to inform PWULA about the study was via a telephone call from members of the orthopaedic team that potential participants already knew through their attendance to the clinic. During the call, the aims of the study and what the participation would involve was explained to the participants. They were informed that their participation was completely voluntary, and that they had the right to withdraw from the study at any time. A convenient time for the face-to-face interviews was arranged and participants were informed that there would be reimbursement for their travel.

### Data collection and analysis

Participants were provided with a copy of the participant information sheet prior to starting the interview. They either read the information themselves or had it read to them. There was an opportunity to ask questions and they were plainly informed that they did not need to continue with the study and had the right to withdraw at any time. To account for all levels of literacy, consent could be provided by completing and signing a written consent form or providing a thumbprint signature.

Due to the exploratory and sensitive nature of the research topic, the use of one-to-one and in-person interviews were felt the most appropriate approach as opposed to telephone or online methods [36]. Following feedback from Phase 1 regarding the importance of participants feeling comfortable with the person interviewing them, a team of four interviewers carried out the interviews (three males and one female with different cultural backgrounds) to provide participants with a choice of the gender and cultural background of their interviewer. The interviews lasted between 30 and 100 minutes, they were audio recorded with consent, transcribed verbatim and translated by a professional company into English followed by a revision performed by two Ugandan members of the research team. Interviews were carried out between February and May 2019.

Assisted by software (QSR International NVivo 12), data was processed and analysed using thematic analysis (Braun & Clarke 2006, 2013). The first stage involved detailed coding of the data to find categories of experiences. Regular meetings allowed a collaborative interpretation of cultural aspects of the coding with the Ugandan researchers. To optimise rigour and consensus, themes were drafted by hand collaboratively through regular online meetings and asynchronous comments on a shared file containing the thematic analysis.

## Results

We have used pseudonyms to protect the identities of the participants. Seven participants identified as female and ten as male. The year of the amputation varied from the 1980’s up to 2018. The cause of the amputation was mostly due to road accidents (nine participants), followed by violence (four participants). Two participants lost their upper limb in a fire and one preferred not to specify the traumatic experience of his limb loss (Table 1). Twelve participants had amputations above the elbow, three below the elbow, one at the elbow and one had amputations of both arms at different levels. Most of the participants did not report to own land and a house, they either rented a house or stayed with family members.

**TABLE 1:** Participants’ characteristics in the following order: pseudonyms, age, gender, cause of amputation, amputation level, amputation side, year of amputation, employment before and after amputation.

Participant pseudonyms	Age (years)	Gender	Aetiology	Amputation level	Year of amputation	Employment before amputation	Employment after amputation	Living situation	Parenting responsibilities
Nabirye	41	Female	Road accident	Above elbow	2016	Fuel tanker driver and Mechanic	Tailoring apprentice	Rent	2 children
Matovu	37	Male	Road accident	Above elbow	1982	Taxi driver	Safari track chaperon and taxi driver	Unspecified, has a wife	Unspecified number of children
Mbabazi	33	Female	Violence	Below elbow	2005	Student and dancer	Pancake seller door to door (past) and shop keeper (present)	Guest in foundation premises	None
Musinguzi	30	Male	Violence	Below elbow	2018	Delivery clerk with national medical stores	Delivery clerk with national medical stores	Alone	None
Namuli	36	Female	Road accident	Above elbow	2018	Laboratory Vector Control Officer	Volunteer school health counsellor	Unspecified, married	1 child
Waiswa	45	Male	Non specified traumatic experience	Shoulder disarticulation one side and above elbow the other side	2013	Cleaner	Plastic bottle collector	Rent, wife left him	6 children, no wife
Nakanjako	42	Female	Road accident	Above elbow	2014	Bananas seller	Bananas seller	Unspecified	2 children
Nalubowa	22	Female	Fire	Above elbow	1997	Not applicable	Student	With sister	None
Opolot	48	Male	Violence	Below elbow	1999	Engineer	Marketing business executive	Unspecified	None
Nantume	22	Female	Road accident	At elbow	2017	Street stall selling juices and hot drinks	Street stall selling juices and hot drinks	Unspecified	1 child
Batte	43	Male	Road accident	Above elbow	2018	Taxi driver	Helping at taxi stand	Owns plot of land with house. With wife and children	Undisclosed number of children
Kakande	68	Male	Illness	Above elbow	2016	Professional soccer player and mechanic	Unemployed	Alone but has a wife	2 children
Kiyimba	27	Male	Road accident	Above elbow	2016	Taxi conductor	Unspecified	With wife and other family members	1 child
Kakuru	36	Male	Road accident	Above elbow	2012	Sim card registerer	Seller	With family members and wife	1 child
Tugume	22	Male	Fire	Above elbow	2005	Not applicable	Undergraduate student	Rent shared room	None
Namara	40	Female	Violence	Above elbow	1999	Street stall selling sweets, cigarettes	Shop keeper	Unspecified	None
Megere	43	Male	Road accident	Above elbow	2016	Farmer	Teacher, piggery keeping	With relatives and wife	7 children

### Thematic analysis

**FIGURE 1:**
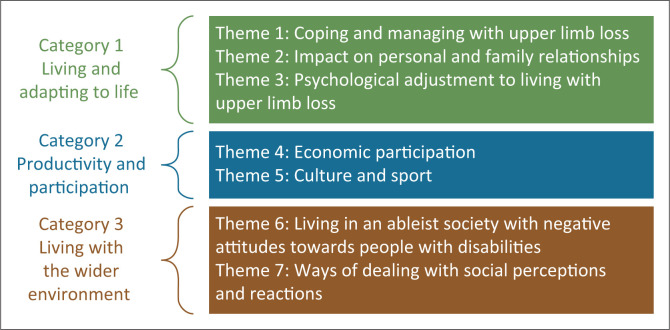
Overview of the three categories of themes. Category 1 Living and adapting to life, Category 2 Participation and productivity and Category 3 Living with the wider environment.

To understand different aspects of the experience of people living with upper limb absence in Uganda, the themes identified were categorised into core categories that illustrate the impact on the individual’s lives. Figure 1 provides an overview of the categories of themes.

#### Category 1: Living and adapting to life

Participants discussed coping techniques after amputation and how it impacted on their personal and family relationships. This also led to descriptions of adjustments to the amputation at a psychological level.

##### Theme 1: Coping and managing with upper limb loss

Participants described practical approaches they had developed to cope while having only one arm. Coping techniques involved using the residual limb (remaining part of the limb on the amputated side), assistive technology, and the support of other people.

Learning to complete activities one-handed often involved learning to incorporate the residual limb:

‘I can sometimes use the remaining part of the limb or the broken side [amputated side] to support when it’s really necessary, but on a larger extent I use my left arm hand, which is okay.’ (Opolot)

Other participants incorporated the use of an assistive device to help complete activities one-handed, for example, to help complete or speed up the task of using jerry cans for bathing. Another means of coping was the use of help from someone else to complete activities. Nakanjako described:

‘I can cook if they have chopped the onions and tomatoes for me, I can cook the sauce but then I can’t use one hand to chop the tomatoes and onions.’

and Kakuru explained: *‘At home I don’t get a lot of challenges because my wife can help me washing my clothes.’*

While Megere had to hire someone:

‘I used to dig, I could dig, I could rear some pigs, I could keep some potatoes and give the pigs the green leaves, but now these days I no longer do it perfectly, so I have to hire people to do it for me.’

Participants gave priority to being able to manage and find new ways of completing daily life activities with one hand. These activities ranged from personal care to holding a phone and farming activities: *‘I can hold a phone with this one and also chop stuff therefore if it can hold a hoe I can dig’ (Matovu).*

For other participants, being able to carry out activities using only one hand was important for the purpose of employment. For example, Matovu who works as a taxi conductor explained how he found a way to manage taking taxi fares using one hand. Despite the innovative ways that participants described managing with their upper limb loss, it was clear that it was still very difficult, and activities took significantly more time to perform and plan, as described by Musinguzi:

‘Though I take much more time, that is what I have learnt, so I usually like to know in advance what I am going to do, to plan and then try to do it in time like everyone else.’

As a student at a university that advocates for PWDs’ rights through having a university funded PWDs organisation, Tugume discusses how meeting fellow PWULA enables him to cope:

‘We share experiences about how we live, about how we can improve our lives, because you may find I use some skill in doing something which my friend doesn’t know […] we share how we can live better in life.’

Most participants did not know what to answer when asked if they needed changes in the social and built environment. Having learned ways to do daily chores with one limb and facing challenges with balance while moving, Kakande described that climbing staircases without handrails is a challenge: *‘There is no building hard for me even when I have one arm, as long as it has these stands [handrails] for climbing and sloping.’*

In contrast, Nakanjako wished her social environment would adjust to be able to express feelings of joy altogether without favouring persons with both hands such as during celebrations:

‘The challenge I get the most – I am catholic woman – even on Sundays, I don’t want to eat food without praying so the challenge I get, we can be around one thousand people or something, my colleagues clap their hands like they do, and I say, “oh my God” me, why me, why?’

##### Theme 2: Impact on personal and family relationships

Participants felt that their upper limb loss had impacted on both personal and family relationships, in terms of the support that they required and their relationship roles. Some descriptions of support were underpinned with a sense of reliance on family members and in some cases feeling a burden. For example, Waiswa, describes feeling like a ‘child’:

‘Because most of the time they just put food on the table, I have to bend myself to the plate to eat that food. Dressing myself is also a challenge, I cannot do it myself. For real am just there like a child.’

For some participants the help received from family members led to feeling a *burden* or over *dependence* on families, regardless of them acknowledging that they were happy to help. These mixed feelings and perceptions about the care received from family members is captured by Namuli:

‘… okay if they are just helping me, let them out of love and not out of being burdened … I was not used to saying things like “hey do this for me” so I get a hard time from that, it is hard, so I find myself very dependable on others.’

and *‘but there are times I feel like, I over burden him.’*

In some cases, shifted family roles had an impact on the participant’s sense of identity; this was described by Matovu, *‘It is very hard, we find it very hard to assure someone that you will be able to look after them when you have one hand.’*

Although several participants were concerned about the amount and impact of the support they were receiving, it was also clear that this support was often viewed positively as comforting and reassuring. Musinguzi said:

‘What comforted me the most were the people around me, I didn’t tell people to come see me, they came, comforted me, supported me and that is one thing I have enjoyed, people around me.”

Batte expressed gratitude to both family and neighbours ‘*she [daughter] has done so much to help me, I have many friends, my neighbours have been helping me if it is washing, I wash.*’ In contrast, Namuli seemed to want to be able to perform certain tasks without support *‘it was bothering us, me, and my husband, he says – but why don’t you no longer call me to scrub you? why don’t you no longer call?’*

##### Theme 3: Psychological adjustment to living with upper limb loss

This theme captures aspects of the psychological response underpinning participants’ ways of adjusting and coping with upper limb loss. This is captured in Mbabazi’s reflections describing how she previously felt about her upper limb loss: *‘I would wake up with the hope of having my hand again, it would kill me every time I wake up […] because I would dream of having it.’* This process of adjustment seemed to be aided by the use of a prosthesis becoming part of her identity, in contrast with it initially being viewed as a burden:

‘It was really a burden, but as time went by, I got used to it, so I know that it is part of me, that is me, I cannot do without it, so I had just to put it in my mind that this is me, so the bad way I looked at has stopped.’ (Mbabazi)

From several participants’ perspectives, an important part of psychological adjustment to living with their limb loss, was the importance of a positive attitude. This was described as a sense of maintaining positive goals, hope and a positive mindset:

‘Some need to know that what happened to them should not stop them from achieving their life goals, I would encourage them to keep their hopes high since life continues.’ (Namara)

And:

‘You know what kills us most is the mindset and if you do not have anything to build your mind in a positive way, you crush slowly’ and ‘the next day family and doctors encouraged me, and I was equally a person with a positive mind, I think I took up their advice.’ (Opolot)

Some participants attributed enduring through the limb loss with the help of their God and friends, for example, Namuli described *‘I have friends, I think this has helped me to move my life in a way, God has helped me when I am with people I am used to.’* Whereas others like Kakuru were able to access counselling from their church leaders:

‘I thought when I got one arm that I will be with one of them, which I see on Kampala streets, begging, but eventually I found myself going to church, pastors helped me with counselling and other people, so as of now I can’t say I can beg.’

#### Category 2: Productivity and participation

Participants described productive and leisure occupations that included participation in culture, sports, community, and employment.

##### Theme 4: Economic participation

Before the amputation, most participants had occupations with long working hours and physical in nature, for example, fuel tanker driver, banana seller, cleaner, street stall seller, farmer, taxi driver and mechanic. After the amputation, some participants were not able or allowed to continue doing the same job (Table 1). The amputation has affected the professional desires of Musinguzi: *‘I am learning to do much of the everyday chaos by myself, so I have put aside some professional goals for now.’* Nabirye is learning tailoring and surviving the day is still tough: *‘I find it very difficult’, ‘I used that [sewing] machine plus my first-born to repair some people’s clothes and get money for rent and feedings.’*

Just as with Nabirye, other participants also struggle to make ends meet daily. Matovu gets on the safari track as a chaperon and said: *‘when we come back, they give like ten thousand and I take it home, that is how we survive, my wife and children.’* While Kakande, being unemployed, reported that *‘when money is nowhere to be seen I buy halves [of medicine prescriptions] so that I can live’* and the living situation affecting his sleep *‘I don’t have peace even at night, I only sleep for four hours in my life.’* In contrast, Mbabazi having had a difficult job of making and selling pancakes, after the amputation now has a job as a shop keeper with significantly better working conditions:

‘I am a bit relaxed because I sleep enough now [laughs] […] with a rested mind, […] not with a stressful mind whereby I know where am I going to be in the morning and who am I going to sell to, instead of will they be bought [pancakes]?, will they not be bought?’

Some participants have experienced discrimination that has affected their employment opportunities. Matovu has unstable employment for this reason: *‘sometimes I work sometimes I do not work, because some people despise you because you are lame, therefore, they cannot hire you.’* While Namuli perceives employers as not wanting to hire her after recurrent messages of *‘we shall call you’, ‘we still need to finish some errands’ and ‘there was restructuring.’* As opposed to Matovu’s friend who encouraged him to concentrate on driving the taxi:

‘We can work together. You can work as a conductor with that one hand. I do not see why you insist on working with the tracks [Safari]. With a track, you make only 10 000 but with a taxi you can make 20 000 shillings every day.’

Coping with caring for children and employment was a hardship before the amputation, as described by Nabirye *‘I used to drive when I was pregnant […]. I used to move with her in the [truck] cabin, driving […] with my baby’.* After the amputation, Nabirye asked doctors *‘will I be able to support myself?’* and wanted to continue taking care of her children *‘I have young people – my children are still young, they still need my help.’* Similarly, Nakanjako cares for two children alone after her husband left her after the amputation: *‘I have to look for what they should eat […] I can’t get from my earnings for school fees for the kids, I am the mother and the father.’*

In contrast, some participants are being cared for by their children, other family members and friends. Waiswa has no wife, and his children help him to get dress in the morning before they leave for school. Nalubowa stays with her sister who supports her to study. Batte’s wife has employment and supports the household economically and Kakande’s friends send him money and *‘the village […] where I grew up and where I work, collect money and send it to me when I am out of money.’* However, the uncertainty of the future caring circumstances is vivid in Waiswa’s words *‘my children are growing older, and time will reach when they leave home and go. Then who will take care of me?’*

##### Theme 5: Culture and sport

Participants described positive and negative experiences of dance, sport, singing and community participation before and after the amputation. Namuli sings and sees this activity as part of her social support system *‘so when I am with people, I am a singer […] gospel, I sing from the choir.’*

Having worked as a dancer in a traditional African dancing company, Mbabazi missed dancing after the amputation, but felt inadequate and undesirable:

‘I really wanted to train because I felt losing my life, I needed my life back, so I went to the national theatre, at first I had it on [a cosmetic prosthetic] so everyone didn’t know what was up with me, then I look around at the kind of dances they do, and then I was like ‘no way’, I didn’t see myself dancing those dances without my limb, […] I realised no dance group is going take me on to dance in there group without a hand.’

Participating in sport was a desirable activity, with some participants actively engaging in it after the amputation, describing sport as a source of wellbeing. Mbabazi jogs in the evenings after work and practises badminton *‘I will stay in badminton forever because that’s where I feel peace of mind, people who understand me, but then out of badminton no one will understand me.’* For Kakande, sport defines his life having been a professional soccer player before the amputation *‘when I wake up early in the morning, I go do exercise because I am a person of exercise.’* For Musinguzi, amputation has not prevented him from participating in sports *‘I used to enjoy swimming and running, and I still do them.’* Tugume practises amputee football and jogging, explaining a typical routine pleasantly ‘*in the evening I go for jogging. Or if I have match, I train amputee football*.’ Whereas Nantume misses participating in netball *‘I really miss active sports participation and the most game that I miss is netball.’*

Apart from sport, dancing and singing being enablers of community participation. Mbabazi described belonging to a rotary, explaining the positive impact *‘they don’t make me feel different, so it is one other thing that has made me grow and develop a high attitude of positive life.’* However, Nabirye and Batte described situations in which discrimination prevented them from participating in their communities. Nabirye said *‘I am isolated in the family plus the community. I feel bad, but I have nothing to do with that’* and Batte explained the contrasting behaviour of friends:

‘I lost some of my friends ever since I lost a limb, sometimes you try calling them and they make your calls busy, others keep telling you that they are not around, yet truthfully, they are dodging you. Yet these were men who we used to socialise […] before I became an amputee.’

#### Category 3: Living within the wider environment

This category captures the experiences of people living with limb loss in the physical and social environment. It includes descriptions of interactions and reactions to peoples responses to disability.

##### Theme 6: Living in an ableist society with negative attitudes towards people with disabilities

Participants described a wide combination of experiences of ableist attitudes towards them. According to the UN definition, ableism is a value system based on functioning, appearance and behaviours that are considered as standard to live a fulfilling life (Special Rapporteur on the Rights of Persons with Disabilities 2019). Ableism is the conception of ableness, of a perfect body with the implication that disability is the loss of ableness (Campbell 2019). Ableism lies in systems of life, personhood and liveability; it is not just a matter of ignorance and negative attitudes towards PWDs (Campbell 2019). In Uganda, participants frequently described experiencing heightened pity; their family members denied their abilities and disregarded their ability to learn with the right support, for example, *‘he [father] presumed that I wouldn’t be able to write’* said Nalubowa. There were also accounts of PWULA being considered outcasts with people withdrawing in shock when greeted with a prosthetic hand and being laughed at. As Musinguzi shared:

‘No matter what you lost, you are still alive and living well, but there are people who don’t want to get near you, who see you as sort of an outcast, but they are not so many, the majority mind their own business or they are just happy for me.’

Participants persistently referred to ‘normality’ while describing their lived experiences. Disabled and non-disabled have the perception that a ‘normal’ person is a non-disabled one, which further reinforces the stigma of disability. In one case the concept of ableism (Campbell 2019) is echoed in a participant’s own view of what is ‘desirable’ in society *‘The strong make with all the capabilities is desirable you get it, it’s nature.’* This perception of normal led Opolot to feel pressured to change the hook type of prosthesis (which is more functional) to a cosmetic one for the benefit of his family in terms of appearing ‘normal’. In addition, his family members were disturbed by the look of the prosthetic device and encouraged Opolot to stop wearing the device. Some PWULA chose not to engage with other PWULA, hinting possible self-stigma *‘I don’t want to feel I belong to the disabled’* (Opolot) while others actively seek connection with PWDs *‘at our village there is a group of disabled persons which I joined although these are people with different disabilities’* (Batte) and are not afraid to say that they are disabled *‘I am disabled’* (Kakande).

Elements of stigma were caused specifically by the association of amputation being a form of punishment, and so PWULA were called names such as ‘thief’, as Matovu explained:

‘Everyone calls you what you are not, that you are a thief, your hand was amputated because you were stealing, that you are lame, all sorts of things like that. All the time you are feeling small.’

Or name calling experienced by Nantume *‘others say am a witch because men are so much interested in me regardless of my disability.’* The term ‘lame’ was associated with disability and used by people toward participants and by participants themselves: *‘even when you go to functions, they always pick on you, “oh there is a lame person”’* (Matovu) and *‘to stay like in the community, they segregate me because now I am a lame person’* (Nabirye). These experiences of discrimination could often be upsetting as Nantume shared:

‘Many discriminate me. Such statements hurt me so much to the extent of asking myself whether for them they use different means of transport that are accident free from the one I used that led me to this.’

Participants described specific interactions and how people reacted when they first noticed their limb absence:

‘There is coldness, there are scenarios when someone buys from you a couple of times but never notices and then when they see it, they ask shockingly “but how come I have never seen you like that?”’ (Nakanjako)

Opolot described how people reacted when they realised that they were wearing a prosthesis leading to the need to persistently explain their limb loss to strangers:

‘It is not a real hand, so he withdraws in shock, then you try to explain, you say – this is a prothesis, I had an accident – and stuff like that, but it went on and on, each time that would happen you had to explain, imagine if you had 10 people like that in a day, it is quite cumbersome.’

It was found that children would often ask the participants about their limb loss and could range from being viewed as being ‘curious’ to a more difficult and distressing situation: *‘challenges that I got were from young kids, they would come not knowing, they call their other friend and stand staring’* (Nakanjako). It was also described that some people even wanted to touch and feel the participant’s residual limb:

‘This part from the bottom above the elbow, when it is merely hanging, someone will see it as having no bone, they desire to touch it, they touch it, they feel it.’ (Namuli)

People’s assumptions that PWULA are not fit, strong or able to work in paid employment were mentioned often. Musinguzi described being asked to show his surgical scars to ‘prove his strength’:

‘They even ask me to remove my shirt, that I can show them what the surgery did, but in public it’s a different …, say most people are not used to seeing an old man very strong but without a limb.’

These assumptions impacted on where participants sought employment and revealed the prejudice by people who did not know them *‘they cannot hire you, therefore, most of the time you find that the people who hire you are those that know you already’* (Matovu). Even if not explicitly stated, there appeared to be a perception by participants themselves that other people would not think they were able to work.

Some PWULA found it difficult to start and preserve intimate relationships. Some participants avoided being in an intimate relationship to focus on themselves after the limb loss, for example Musinguzi explains:

‘It’s very hard and I am focusing on is improving my own life, so I have shut the door to relationships for now. I would like to have a nice time if I ever marry. When you don’t have a piece of something to bring that strength, you are not so desirable.’

Matovu also discussed how they had to assure a potential partner that their limb loss would not affect their ability to care for them:

‘It is very hard to assure someone that you will be able to look after them when you have one hand. To look after her in everything, that is the hardest thing.’

Waiswa explained how his relationship had ended because of his limb loss, *‘when I got the accident, my wife ran away from me and left me with 6 children.’* Some of the female participants described being eager to date but faced psychological insecurity or lack of self-confidence:

‘Guys come to date you, and you have one limb, and you are putting on a long-sleeved shirt, he feels confident talking to you. However, much you fight for the right thing and rights, there are things you cannot take away from the mind, so that is why you see so many people that are physically challenged have kids, but they have no husbands.’ (Mbabazi)

Other participants provided more specific details of the experiences and difficulties they had faced in maintaining relationships:

‘For the last 20 years since I lost my limb, several men have approached me intending just to sleep with me but not to marry, many of them, when you move out with them, they tend to keep a distance as if I am not of the class they should love. Yet, behind doors they pretend to love me. Whenever I witness that I also distance myself and get out of such relationships. Others could even promise to recruit a housemaid to help me with housework, but I still refused after going through the experience of the first two men […]. So deep inside my mind, men do such to me because I am a person with an upper limb loss.’ (Namara)

##### Theme 7: Ways of dealing with social perceptions and reactions

This theme presents the ways of reacting or changing behaviour to avoid or manage ableism (Campbell 2019). For example, through self-segregation, attempting to hide their disability or seeking to empower their disabled identity despite the hurdle.

A key part of managing social perceptions was self-segregation (voluntarily avoiding a range of social situations), as explained by Batte *‘I used to go to my relatives and friends’ homes for social events, but I am no longer feeling comfortable to go there.’* This avoidance was also due to not being able to cope outside of their home without their typical sources of support, *‘because who will be there to feed me when that moment of eating food reaches? So, I rather not go there’* (Waiswa). In contrast, Mbabazi reacted with a positive and empowering approach:

‘Especially for us, I wasn’t born this way, so I have to make sure I fight, that’s why I always have to put it on [the prosthesis], like to fight that stigma.’

Participants described how they intentionally covered up their limb absence to avoid interactions. Covering with a scarf or long sleeves was used regardless of gender. Nabirye explains how she wore a scarf all the time regardless of the weather or time of the day:

‘It is difficult for me and at times I keep myself inside the house. Even though I come outside like this, I can’t come outside without a scarf. Even if it is summer or winter, I must cover myself, even if it’s night I have to cover myself because of that.’

Nantume felt frustrated from having to cover her limb loss: *‘I am tired of wrapping up myself with this scarf on this side where I lost the limb.’* Being a driver, Batte covered his limb loss to try to make clients feel ‘safe’ and to hide the upper limb loss:

‘I drive using the remaining upper limb, but I try so much not for passengers to notice that the person driving them has one limb’ and added ‘passengers will also be feeling safe. I will only need to wear a long-sleeved shirt to cover the prosthesis properly, such that it cannot easily be identified by passengers.’

Several participants believed that an important function of wearing a prosthesis was to cover up their limb absence or disability. Namara discusses how having access to a prosthesis would hypothetically eliminate the need to wear a scarf to cover up her limb loss:

‘Most people who do not know that I am missing a limb, keep asking why I need the scarf on. So, if I manage to get a prosthesis, I believe I will be able to look smart in public and will not need a scarf.’

Mbabazi discussed hiding the disability as the main reason for wearing her prosthesis:

‘Actually the biggest fear was the first time they were finding out that I don’t have my second hand, it really bothered me a lot, so I think that’s the biggest reason I had to put it on, whether it is hot or rainy or heavy or am sick. I had to put it on because I want them not to feel like they have to make me the centre of attention.’

Participants emphasised the importance of being seen with having both arms, as explained by Kiyimba *‘actually if you wear it on, someone will see as if you are, having two limbs.’*

## Discussion

The contribution of this study is addressing for the first time the lived experience of PWULA living in Kampala, Uganda, which lacks in the literature. From the individual accounts, this paper contributes a picture of what it means to live with an upper limb amputation in Uganda, a minority community with a disability that has remained mostly under supported and understudied by the disability research community and corresponding literature. This paper presents three main findings: (1) PWULA need psychological and occupational support services which are not available in Uganda, (2) PWULA want to work but face multiple barriers to employment and limited support, this is combined with complex parenting and caring responsibilities (3) the local Ugandan culture and social structures affect in both positive and negative ways the everyday life of PWULA.

Participants described experiences of discrimination, stigma, stereotypes, and prejudice or feeling stereotyped, which has been discussed in an opinion paper and in a report within sub-Saharan Africa (Etieyibo & Omiegbe 2016; Rohwerder 2018). In this qualitative work, participants shared experiences of discrimination, which appeared to be in favour of able-bodied people (ableism), ubiquitous notions of normality-abnormality. These verbal and non-verbal discriminatory behaviours and interrogative gestures were described as explicit forms of unwanted attention that PWULA had experienced. Although prosthetic provision is precarious and not necessarily fit for purpose, when PWULA are able to access a prosthesis, it is mainly used to hide disability. Although there is limited comparable research exploring the views of PWULA in low and middle income countries (LMICs), in this study the management of ableism by PWULA is centred in evasive manoeuvres, such as evading socialising and seeking physical isolation. Despite the detailed experiences of a wide range of events, it was found that none of the participants reported confronting responses to innocuous and hostile attitudes towards them nor attempting to affirm their minority identity. No participant reported actively seeking to dismantle the ableist customs. In this study, it was shown that discrimination appeared to impact on various lived experiences, including support post amputation, to preserve or find employment, to preserve family relationships, to establish intimate relationships, to obtain respectful recognition from the wider community and to participate in their community. This appears to be similar to a growing body of qualitative research exploring the experiences of individuals with lower limb absence, who described that the key function of wearing a lower prosthesis was to appear ‘non’ or ‘less-disabled’ and capable of being able to work, as the use of crutches would highlight their disability and affect being able to gain employment (Ennion & Manig 2019; Kam et al. 2015; Ramstrand et al. 2021; Stuckey et al. 2020). In these studies that focussed on lower limb absence, it was shown that prosthesis use was related to increased self-worth and value in terms of being able to engage in society, which was compared to increased dependence on other people prior to prosthesis use (Ennion & Manig 2019; Ramstrand et al. 2021). This perhaps links to the concept of ‘prosthetically enabled identities’ developed through a qualitative synthesis process (Murray & Forshaw 2013) and highlights the role of prosthesis in regaining identities, adjusting to limb loss and enabling new identities (Järnhammer et al. 2018).

Experiencing hardship while making ends meet was expressed as a challenge by many participants in this study, especially those with dependants. Past work has noted that with the right support such as a professional multidisciplinary team through the coping phase, a growth mindset and a strong supporting community could help to change the negative perception that PWULA have of disability (McDonald et al. 2020). Within our study we saw examples of the power of belonging and positive identity traits when participants took part in sports, art, and worship.

A study with PWDs from three African countries, namely, Kenya, Uganda and Zambia showed that empowering PWDs through education and removing barriers such as discrimination at places of work promotes economic success (Shakespeare et al. 2019) and that PWULA can perceive their ability positively (McDonald et al. 2020). Various participants in this study are struggling to find and secure employment and they do not have access to support. This lack of support is contrary to the Ugandan Persons with Disabilities Act (The Republic of Uganda 2019) and against equity (United Nations 2006). Only a minority of participants have successful support networks for employment, found within their close social relations (close friends and family). Some participants are struggling parents and have additional caring responsibilities (extended family). These participants highlighted the experience of viewing life as difficult and experiencing psychological stress when managing parenting and employment responsibilities. Few participants have found suitable and/or healthy employment or someone to take care of them economically after their amputation.

Participants also reported taking time to cope with the life-changing event of limb loss, heal or adjust to both the physical and psychological trauma and adapt to their new bodies, echoing results from studies about the coping trajectory in PWDs (Caddick et al. 2019; Horgan & MacLachlan 2004). Consistent with (Daniele et al. 2004), we found that PWULA who had lost their dominant hands found it particularly hard to adjust to the time it took them to perform tasks. A loss of a dominant hand correlates positively with the delay in response while examining the effect of limb loss during mental simulation of body-part movements (Daniele et al. 2004). In addition, loss of a dominant limb increases the errors made while performing a task (Daniele et al. 2004). Various participants are actively engaging in recreational activities, which are important components for good health and an important domain of functioning in life that is relevant to health and disability (World Health Organization 2001, 2012).

It has been identified that in Uganda some women are reluctant to accept help from men and some men are ill-treated if they engage in domestic work (Guloba et al. 2018). Thus, if the female partner experiences an upper limb amputation, it directly affects the gender-based dynamic. Even when male partners are willing and able to adapt, some female participants insisted on preserving the expected socio-cultural norm, regardless of how challenging this is with only one arm and no access to prosthetics or additional support at home. In this study, participants also highlighted the importance and impact of their prosthesis on establishing and maintaining intimate relationships. Similar findings were reported in a systematic review of 11 studies exploring the relationship between sexuality and amputation, where it was found that there was an impact of the amputation of a limb on sexuality and sexual function to some degree across all studies (Geertzen, Van Es & Dijkstra 2009). However, it is important to note that some of the studies are quite dated now (for example, ranging from 1945 to 2002), were predominantly carried out in higher income settings, and relate mainly to lower limb loss. Other than intimate relationships, PWULA find motivation in friends, family members, religion, engaging in recreational or leisure activities and their own self-esteem. However, 15 out of 17 of our participants did not actively seek support from, or to support other PWDs. Previous research has highlighted the importance of psychosocial factors within the adjustment of PWULA to limb loss (Klarich & Brueckner 2014; Thomas & Siller 1999; Wald & Alvaro 2004); many PWULA seemed to not have undergone this important process of coping and adapting to living with their limb loss (Murray & Forshaw 2013). This was often described by some participants as a belief that *socialising with PWDs would make them disabled*, indicating that some PWULA have internalised ableism as explored by Campbell (2008). The accounts of PWULA in Uganda seem accumulative and recurring experiences of ableism pervading their self-awareness, resulting in distancing PWDs from each other while emulating the wider community’s ableist norms (Campbell 2008).

### Recommendations for PWULA, non-governmental organisations and the Ugandan government

Past work exploring specific social and cultural issues at end user and wider stakeholder levels exists for lower limb prosthetics and has been noted that such factors are often overlooked in the design of prosthetic devices (Kam et al. 2015). This study is an invitation to the government and local organisations to help PWULA to actively raise their voice through supporting the creation of DPOs specific to PWULA. Without the transformative participation of PWDs (White 1996) to gather evidence, policy and practice have no transformative impact. We also suggest that new partnerships are needed between the government and local organisations to help combat ableism through country wide unified activities that consider multiple religions, ethnicity, and tribalism. Learnings from mental health and psychosocial support in culturally diverse Ugandan refugee camps may be useful (Musiimenta, Miles & Murakami 2020). In addition, past work has shown how academia and disabled people organisations can perform collaborative research in African countries like Liberia, Kenya, Uganda and Sierra Leone (Kett et al. 2019). Academics and government staff who hold decision making powers would benefit from specialist knowledge on DPOs on research methodologies to gather evidence (Kett et al. 2019).

### Implications for policy development

Uganda ratified the United Nations Convention on the Rights of Persons with Disabilities and in 2016 reported to the UN Committee who gave a list of concerns and recommendations (Committee on the Rights of Persons with Disabilities 2016). Some of the concerns relevant to the Findings of this study were as follows: use of derogatory language towards PWDs, absence of mechanisms to consult DPOs beyond the National Council for Disability, insufficient legal measures to protect PWDs against discrimination and lack of mechanisms to create public awareness of stigmatising cultural practises. Another study in 2010 also made similar recommendations to improve legislation and services for PWDs in Uganda (Millward et al. 2005). The experiences shared by PWULA in this study indicate that many such concerns identified in 2010 and 2016 have not been resolved. Furthermore, in 2019, policy and strategy documents produced by the African Union were analysed (Lang et al. 2019) and showed that recognition of the rights of PWDs is not integrated within implementation plans, budgetary allocations, enforcement mechanisms and disaggregated management information systems. Thus, we recommend Ugandan stakeholders to increase the efforts to fulfilling the recommendations by the committee on the rights of persons with disabilities from 2016, especially those related to understanding PWDs, in preparation for the next periodic report, due in October 2022.

## Strengths and limitations

Consistent with literature from other LMICs, road accidents was the leading cause for amputations among the participants engaged in this study (Bezerra de Sousa et al. 2017). The limb loss was violent and traumatic for some participants and the details of their experiences is beyond the scope of this article.

The findings of this study are a unique insight into the lives of 17 participants and should be viewed as a starting point for future research and understanding of this issue. This study was focussed on Kampala, which complements past research looking at the experiences of PWULA living in northern Uganda (Atim et al. 2020; Okello et al. 2019).

One challenge of research in LMIC’s settings is that the expectations of participants need to be managed throughout and after the study (Chesser, Porter & Tuckett 2020). Participants had high expectations that had to be clarified throughout this study.

## Recommendations for future research

The findings from both phases of this research programme will inform design requirements (Kenney et al. 2018). We encourage other countries to perform similar studies to increase evidence of prosthetics needs, so policy makers can speak for PWULA and help develop prosthetic services.

## Conclusion

The lived experience of people with upper limb absence has been understudied in the literature. The contribution of this study is the documentation of the experiences of PWULA living in Uganda. We found that those experiences are predominantly negative and are comparable to the concerns that the UN Committee on the Rights of People with Disabilities already brought with Ugandan authorities in 2016. The recommendations of such a committee appear to have not been implemented or not impacted or not reached yet people with upper limb absence. Complex challenges require partnerships and multiple stakeholders, including policymakers and action by the ministry of health. Uganda would benefit from a wide campaign to dismantle ableism, support the formation of Disabled Peoples Organisations and the establishment of appropriate services for people with upper limb absence.

## Footnotes

1 Intersectionality: initially a legal concept coined by Kimberle Crenshaw in 1989 and defined as ‘a prism to bring to light dynamics within discrimination law that weren’t being appreciated’. https://chicagounbound.uchicago.edu/cgi/viewcontent.cgi?article=1052&context=uclf

2 Multidimensional poverty: poverty in all its forms and dimensions as declared by the United Nations in 2015 through the Transforming our world: the 2030 agenda for sustainable development A/RES/70/1.

## References

[CIT0001] Ajibade, A., Akinniyi, O.T. & Okoye, C.S., 2013, ‘Indications and complications of major limb amputations in Kano, Nigeria’, *Ghana Medical Journal* 47(4), 185–188. 24669024 PMC3961849

[CIT0002] Armstrong, T.W., Williamson, M.L.C., Elliott, T.R., Jackson, W.T., Kearns, N.T. & Ryan, T., 2019, ‘Psychological distress among persons with upper extremity limb loss’, *British Journal of Health Psychology* 24(4), 746–763. 10.1111/bjhp.12360 30941874

[CIT0003] Atim, P., Loum, C.S., Okello, T.R., Magada, S.M., Yagos, W.O., Abelle, P. et al., 2020, ‘Prevalence of Major Limb Loss (MLL) in post conflict Acholi sub-region of Northern Uganda: Cross sectional study’, *bioRxiv* 2020.05.14.095836. 10.1101/2020.05.14.095836

[CIT0004] Baker, A.C. & Clouse, W.D., 2016, ‘14 – Upper extremity and junctional zone injuries’, in T.E. Rasmussen & N.R.M. Tai (eds.), *Rich’s vascular trauma*, 3rd edn., Elsevier, Philadelphia, PA.

[CIT0005] Beisland, L.A. & Mersland, R., 2014, ‘Staff characteristics and the exclusion of persons with disabilities: Evidence from the microfinance industry in Uganda’, *Disability & Society* 29(7), 1061–1075. 10.1080/09687599.2014.902362

[CIT0006] Bezerra de Sousa, L.R., Santos de Sousa, G., Muradas da Costa Monroe, K.C. & Silva Pereira, M.G., 2017, ‘Injury accident reporting at a public hospital in the Brazilian Amazon’, *Revista Brasileira em Promocao da Saude* 30(1), 64–71. 10.5020/18061230.2017

[CIT0007] Braun, V. & Clarke, V., 2006, ‘Using thematic analysis in psychology’, *Qualitative Research in Psychology* 3(2), 77–101. 10.1191/1478088706qp063oa

[CIT0008] Braun, V. & Clarke, V., 2013, *Successful qualitative research: A practical guide for beginners*, Sage, London.

[CIT0009] Caddick, N., Cullen, H., Clarke, A., Fossey, M., Hill, M., Mcgill, G. et al., 2019, ‘Ageing, limb-loss and military veterans: A systematic review of the literature’, *Ageing and Society* 39(8), 1582–1610. 10.1017/S0144686X18000119

[CIT0010] Campbell, F.A.K., 2008, ‘Exploring internalized ableism using critical race theory’, *Disability & Society* 23(2), 151–162. 10.1080/09687590701841190

[CIT0011] Campbell, F.K., 2019, ‘Precision ableism: A studies in ableism approach to developing histories of disability and abledment’, *Rethinking History* 23(2), 138–156. 10.1080/13642529.2019.1607475

[CIT0012] Chesser, S., Porter, M.M. & Tuckett, A.G., 2020, ‘Cultivating citizen science for all: Ethical considerations for research projects involving diverse and marginalized populations’, *International Journal of Social Research Methodology* 23(5), 497–508. 10.1080/13645579.2019.1704355

[CIT0013] Commission on the Social Determinants of Health, 2008, *Closing the gap in a generation: Health equity through action on the social determinants of health*, Final Report of the Commission on Social Determinants of Health, World Health Organization, Geneva.

[CIT0014] Committee on the Rights of Persons with Disabilities, 2016, *Concluding observations on the initial report of Uganda*, United Nations, Geneva.

[CIT0015] Daniele, N., Daprati, E., Rigal, F., Parsons, L. & Sirigu, A., 2004, ‘Left and right hand recognition in upper limb amputees’, *Brain* 127(1), 120–132. 10.1093/brain/awh006 14607796

[CIT0016] Davidson, J.H., Khor, K.E. & Jones, L.E., 2010, ‘A cross-sectional study of post-amputation pain in upper and lower limb amputees, experience of a tertiary referral amputee clinic’, *Disability and Rehabilitation* 32(22), 1855–1862. 10.3109/09638281003734441 20345252

[CIT0017] Desmond, D.M. & Maclachlan, M., 2010, ‘Prevalence and characteristics of phantom limb pain and residual limb pain in the long term after upper limb amputation’, *International Journal of Rehabilitation Research* 33(3), 279–282. 10.1097/MRR.0b013e328336388d 20101187

[CIT0018] Dillon, M.P., Fatone, S., Ramstrand, N. & Hafner, B.J., 2019, ‘Prosthetics and Orthotics International welcomes qualitative research submissions’, *Prosthetics and Orthotics International* 43(4), 366–368. 10.1177/030936461986392232102604

[CIT0019] Dolan, C., 2009, *Social torture: The case of Northern Uganda, 1986–2006*, Berghahn Books.

[CIT0020] Eide, A.H., Khupe, W. & Mannan, H., 2014, ‘Development process in Africa: Poverty, politics and indigenous knowledge’, *African Journal of Disability* 3(2), a75. 10.4102/ajod.v3i2.75 PMC5442507 28730008

[CIT0021] Ennion, L. & Manig, S., 2019, ‘Experiences of lower limb prosthetic users in a rural setting in the Mpumalanga Province, South Africa’, *Prosthetics and Orthotics International* 43(2), 170–179. 10.1177/0309364618792730 30112980

[CIT0022] Etieyibo, E. & Omiegbe, O., 2016, ‘Religion, culture, and discrimination against persons with disabilities in Nigeria’, *African Journal of Disability* 5(1), 192–192. 10.4102/ajod.v5i1.192 28730043 PMC5433448

[CIT0023] Geertzen, J.H.B., Van Es, C.G. & Dijkstra, P.U., 2009, ‘Sexuality and amputation: A systematic literature review’, *Disability and Rehabilitation* 31(7), 522–527. 10.1080/09638280802240589 19117187

[CIT0024] Guloba, M., Katunze, M., Ssewanyana, S., Ahikire, J., Musiimenta, P., Boonabaana, B. et al., 2018, *Gender roles and the care economy in Ugandan households – The case of Kaabong, Kabale and Kampala Districts*, Oxfam GB for Oxfam International, Oxford, UK.

[CIT0025] Hayes, H., Buckland, S. & Tarpey, M., 2012, *Briefing notes for researchers: Public involvement in NHS, public health and social care research*, INVOLVE, National Institute for Health Research.

[CIT0026] Horgan, O. & Maclachlan, M., 2004, ‘Psychosocial adjustment to lower-limb amputation: A review’, *Disability and Rehabilitation* 26(14–15), 837–850. 10.1080/09638280410001708869 15497913

[CIT0027] Järnhammer, A., Andersson, B., Wagle, P.R. & Magnusson, L., 2018, ‘Living as a person using a lower-limb prosthesis in Nepal’, *Disability and Rehabilitation* 40(12), 1426–1433. 10.1080/09638288.2017.1300331 28320228

[CIT0028] Johansen, H., Trine, B., Andersen, L.Ø., Rand-Hendriksen, S. & Østlie, K., 2018, ‘Education and work participation among adults with congenital unilateral upper limb deficiency in Norway: A cross-sectional study’, *PLoS One* 13(12), e0207846. 10.1371/journal.pone.0207846 30540806 PMC6291096

[CIT0029] Kam, S., Kent, M., Khodaverdian, A., Daiter, L., Njelesani, J., Cameron, D. et al., 2015, ‘The influence of environmental and personal factors on participation of lower-limb prosthetic users in low-income countries: Prosthetists’ perspectives’, *Disability and Rehabilitation: Assistive Technology* 10(3), 245–251. 10.3109/17483107.2014.905643 24694038

[CIT0030] Kenney, L., Ssekitoleko, R., Chadwell, A.E.A., Donovan-Hall, M., Morgado Ramirez, D.Z., Holloway, C. et al., 2019, ‘Prosthetic service in Uganda – A series of studies to inform the design of a low cost, but fit-for-purpose, body powered prosthesis’, in N. Layton & J. Borg (eds.), *Global perspectives on assistive technology: Proceedings of the great consultation*, pp. 414–426, World Health Organization, Geneva.

[CIT0031] Kenney, L., Ssekitoleko, R., Mwaka, E., Donovan-Hall, M., Morgado Ramirez, D.Z., Chadwell, A.E.A. et al., 2018, ‘Upper-limb prostheses for low- and middle income countries’, *IPEM Scope* 27, viewed 22 February 2022, from http://usir.salford.ac.uk/id/eprint/52732/8/SCOPE_December2018_LR.pdf.

[CIT0032] Kett, M., Carew, M.T., Asiimwe, J.-B., Bwalya, R., Gitonga, A., Nyehn, B.A. et al., 2019, ‘Exploring partnerships between academia and disabled persons’ organisations: Lessons learned from collaborative research in Africa’, *IDS Bulletin* 50(1), 65–78. 10.19088/1968-2019.106

[CIT0033] Klarich, J. & Brueckner, I., 2014, ‘Amputee rehabilitation and preprosthetic care’, *Physical Medicine and Rehabilitation Clinics of North America* 25(1), 75–91. 10.1016/j.pmr.2013.09.005 24287241

[CIT0034] Lang, R., Schneider, M., Kett, M., Cole, E. & Groce, N., 2019, ‘Policy development: An analysis of disability inclusion in a selection of African Union policies’, *Development Policy Review* 37(2), 155–175. 10.1111/dpr.12323

[CIT0035] Lao, C., Seghers, F., Savage, M., End Fineberg, A., Goedde, B., Austin, V. et al., 2020, *Product narrative: Prostheses*. A market landscape and strategic approach to increasing access to prosthetic devices and related services in low- and middle-income countries, ATscale under the AT2030 Programme.

[CIT0036] Ligthelm, E.J. & Wright, S.C.D., 2014, ‘Lived experience of persons with an amputation of the upper limb’, *International Journal of Orthopaedic and Trauma Nursing* 18(2), 99–106. 10.1016/j.ijotn.2013.08.018

[CIT0037] Maduri, P. & Akhondi, H., 2020, *Upper limb amputation*, StatPearls Publishing LLC, Treasure Island, FL. 31082006

[CIT0038] Mcdonald, C.L., Bennett, C.L., Rosner, D.K. & Steele, K.M., 2020, ‘Perceptions of ability among adults with upper limb absence: impacts of learning, identity, and community’, *Disability and Rehabilitation* 42(23), 3306–3315. 10.1080/09638288.2019.1592243 30999780

[CIT0039] Millward, H., Ojwang, V.P., Carter, J.A. & Hartley, S., 2005, ‘International guidelines and the inclusion of disabled people. The Ugandan story’, *Disability & Society* 20(2), 153–167. 10.1080/09687590500059101

[CIT0040] Muhumuza, M.F. & Bangirana, A., 2015, *Indicators and the changing pattern of amputations at a tertiary hospital in Uganda. A 10-year retrospective study*, ResearchGate. https://doi.org/10.13140/RG.2.1.4508.7841. https://www.researchgate.net/publication/279534665_INDICATIONS_AND_THE_CHANGING_PATTERN_OF_AMPUTATIONS_AT_A_TERTIARY_HOSPITAL_IN_UGANDA_A_10-YEAR_RETROSPECTIVE_STUDY.

[CIT0041] Murphy, J.W., 2005, ‘Social norms and their implications for disability’, *Journal of Social Work in Disability & Rehabilitation* 4(1–2), 153–163. 10.1300/J198v04n01_09

[CIT0042] Murray, C.D. & Forshaw, M.J., 2013, ‘The experience of amputation and prosthesis use for adults: A metasynthesis’, *Disability and Rehabilitation: An International, Multidisciplinary Journal* 35(14), 1133–1142. 10.3109/09638288.2012.723790 23033871

[CIT0043] Musiimenta, C., Miles, B. & Murakami, N.J., 2020, ‘“We still have tribalism in the camp”: Navigating ethnic conflict in a psychosocial support group’, *Social Work with Groups* 43(1–2), 39–45. 10.1080/01609513.2019.1638644

[CIT0044] Okello, T.R., Magada, S.M., Atim, P., Ezati, D., Campion, A., Moro, E.B. et al., 2019, ‘Major limb loss (MLL): An overview of etiology, outcomes, experiences and challenges faced by amputees and service providers in the post-conflict period in Northern Uganda’, *Journal of Global Health Reports* 3, e2019028. 10.29392/joghr.3.e2019028

[CIT0045] Ramstrand, N., Maddock, A., Johansson, M. & Felixon, L., 2021, ‘The lived experience of people who require prostheses or orthoses in the Kingdom of Cambodia: A qualitative study’, *Disability and Health Journal* 14(3), 101071. 10.1016/j.dhjo.2021.101071 33583726

[CIT0046] Rohwerder, B., 2018, *Disability stigma in developing countries*, K4D Helpdesk Report, Knowledge, evidence and learning for development, Institute of Development Studies, Brighton, UK.

[CIT0047] Shahsavari, H., Matourypour, P., Ghiyasvandian, S., Ghorbani, A., Bakhshi, F., Mahmoudi, M. et al., 2020, ‘Upper limb amputation; Care needs for reintegration to life: An integrative review’, *International Journal of Orthopaedic and Trauma Nursing* 38, 100773. 10.1016/j.ijotn.2020.100773 32362398

[CIT0048] Shakespeare, T., Mugeere, A., Nyariki, E. & Simbaya, J., 2019, ‘Success in Africa: People with disabilities share their stories’, *African Journal of Disability* 8, 522. 10.4102/ajod.v8i0.522 31049311 PMC6489159

[CIT0049] Sood, S., Kostizak, K., Stevens, S., Cronin, C., Ramaiya, A. & Paddidam, P., 2020, ‘Measurement and conceptualisation of attitudes and social norms related to discrimination against children with disabilities: A systematic review’, *International Journal of Disability, Development and Education* 1–16. 10.1080/1034912X.2020.1786022

[CIT0050] Special Rapporteur on the Rights of Persons with Disabilities, 2019, *Report on the impact of ableism in medical and scientific practice*, United Nations, Geneva.

[CIT0051] Staats, T.B., 1996, ‘The rehabilitation of the amputee in the developing world: A review of the literature’, *Prosthetics and Orthotics International* 20(1), 45–50. 10.3109/03093649609164415 8740077

[CIT0052] Stuckey, R., Draganovic, P., Ullah, M.M., Fossey, E. & Dillon, M.P., 2020, ‘Barriers and facilitators to work participation for persons with lower limb amputations in Bangladesh following prosthetic rehabilitation’, *Prosthetics and Orthotics International* 44(5), 279–289. 10.1177/0309364620934322 32686604

[CIT0053] Stutts, L.A., Bills, S.E., Erwin, S.R. & Good, J.J., 2015, ‘Coping and posttraumatic growth in women with limb amputations’, *Psychology, Health & Medicine* 20(6), 742–752. 10.1080/13548506.2015.1009379 25661248

[CIT0054] The Republic of Uganda, 2019, *The Persons with Disabilities Act*, Parliament, Uganda.

[CIT0055] Thomas, K. & Siller, J., 1999, ‘Object loss, mourning, and adjustment to disability’, *Psychoanalytic Psychology* 16(2), 179–197. 10.1037/0736-9735.16.2.179

[CIT0056] Uganda Bureau of Statistics, 2018, *Uganda functional difficulties survey 2017*, Indicators report, Kampala.

[CIT0057] Uganda Bureau of Statistics, 2019, *Persons with disabilility – Bridging the gap through statistics*, Thematic series based on The National Population and Housing Census 2014, Kampala.

[CIT0058] United Nations, 2006, *Convention on the Rights of Persons with Disabilities (CRPD)*, Department Of Economic And Social Affairs, Geneva.

[CIT0059] United Nations Development Programme & Oxford Poverty and Human Development Initiative, 2019, *The 2019 global Multidimensional Poverty Index (MPI) – Illuminating inequalities*, United Nations Development Programme and Oxford Poverty and Human Development Initiative.

[CIT0060] Vargas, M.A.D.O., Ferrazzo, S., Schoeller, S.D., Drago, L.C. & Ramos, F.R.S., 2014, ‘The healthcare network to the amputee/Rede de atenção à saúde à pessoa amputada’, *Acta Paulista de Enfermagem* 27(6), 526–532. 10.1590/1982-0194201400086

[CIT0061] Wald, J. & Alvaro, R., 2004, ‘Psychological factors in work-related amputation: Considerations for rehabilitation counselors’, *Journal of Rehabilitation* 70, 6–15.

[CIT0062] White, S.C., 1996, ‘Depoliticising development: The uses and abuses of participation’, *Development in Practice* 6(1), 6–15. 10.1080/0961452961000157564

[CIT0063] Woods, L., Hevey, D., Ryall, N. & O’keeffe, F., 2018, ‘Sex after amputation: The relationships between sexual functioning, body image, mood and anxiety in persons with a lower limb amputation’, *Disability and Rehabilitation* 40(14), 1663–1670. 10.1080/09638288.2017.1306585 28359182

[CIT0064] World Health Organization, 2001, *International classification of functioning, disability and health*, World Health Organization, Geneva.

[CIT0065] World Health Organization, 2012, *Measuring health and disability: Manual for WHO Disability Assessment Schedule (WHODAS 2.0)*, World Health Organization, Geneva.

